# Supplementary material for: Thermodynamics of organic electrochemical transistors
Source: Nat Commun. 2022 Aug 3;13:4514. doi: 10.1038/s41467-022-32182-7 (PMC9349225; doi:10.1038/s41467-022-32182-7)
Supplement: Supplementary file 1 — Supplementary Information [file 41467_2022_32182_MOESM1_ESM.pdf]

Supplementary Information

Thermodynamics of Organic Electrochemical Transistors

Matteo Cucchi[1,2*], Anton Weissbach[2], Lukas M. Bongartz[2], Richard Kantelberg[2], Hsin Tseng [2], Hans Kleemann[2], Karl Leo[2]
[1] Laboratory for Soft Bioelectronic Interfaces, Neuro-X Institute, École Polytechnique Fédérale de Lausanne (EPFL), Geneva, Switzerland
[2] Technische Universität Dresden, Dresden, Germany

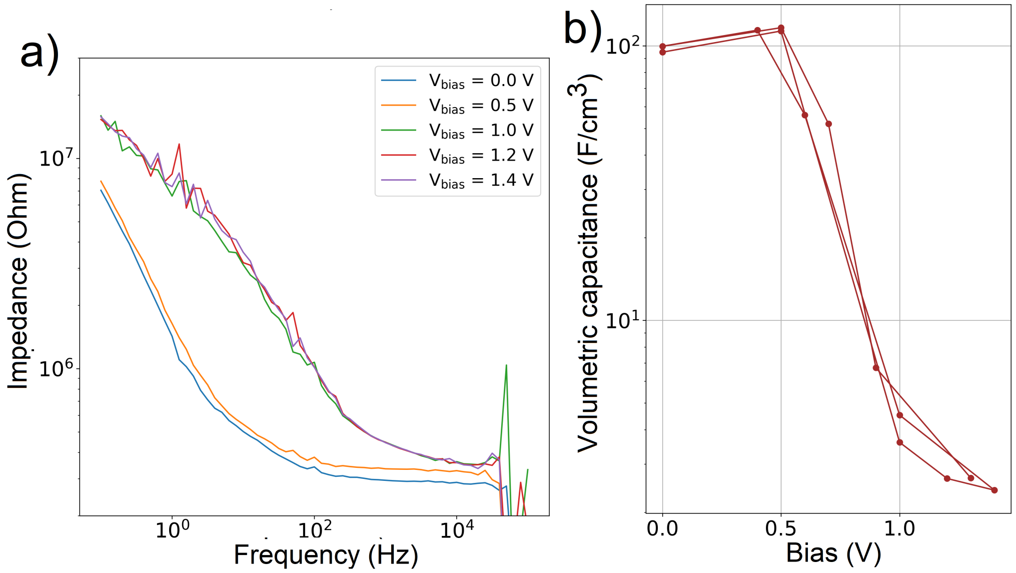

Supplementary Figure 1: **Gate-dependent capacitance of OECTs**. a) electrochemical impedance spectroscopy of an OECT channel (working electrode) (200 μm x 50 μm x 100 nm) measured with an Ag/AgCl electrode (reference and counter electrode) with amplitude of 10 mV and varying bias. b) the extracted channel capacitance, fitted with a Randles cell, showing that the capacitance of the channel is not fixed, rather strongly gate-dependent.

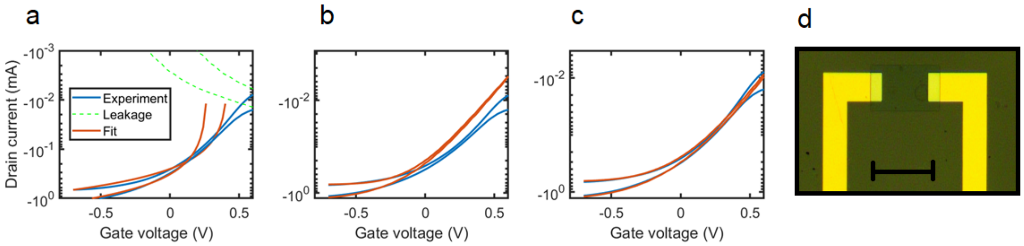

Supplementary Figure 2: **Transfer curves and fits**. The curves plotted in linear scale in Fig. 2 are here reported in logarithmic scale and fitted with the a) the Bernards model, b) the thermodynamic model with the entropic term only and c) including the enthalpic term. Panel d) shows a micrograph of the device (scale bar 200 μm).

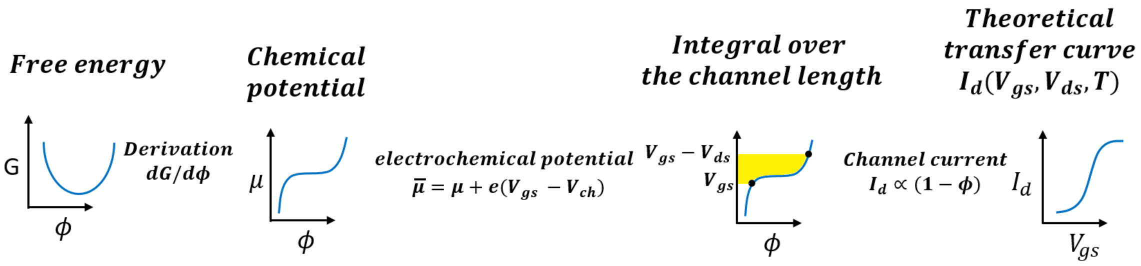

Supplementary Figure 3: **Sketch of the mathematical treatment proposed in the main text, from Gibbs free energy to the transfer curve.**

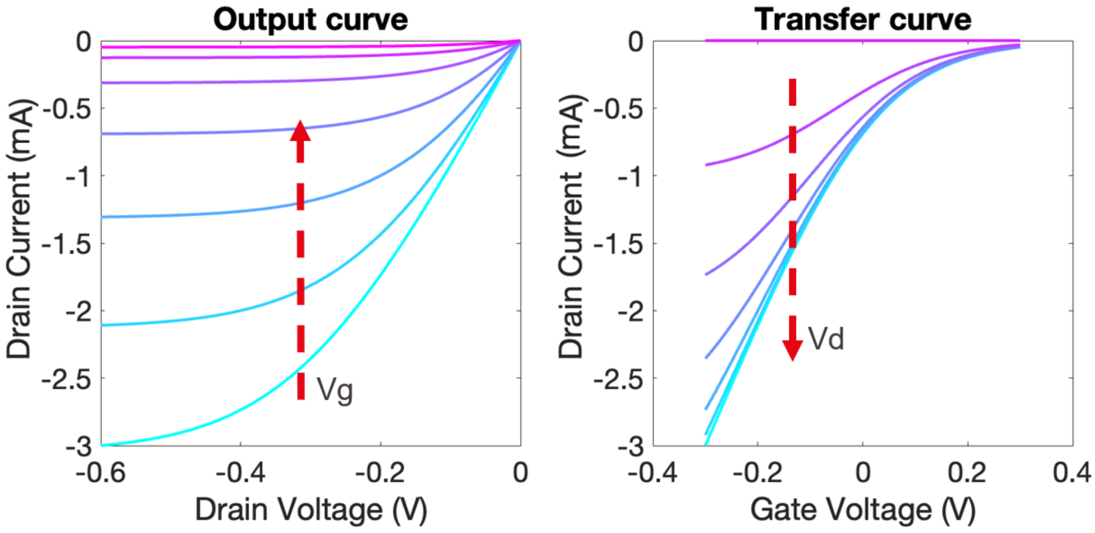

Supplementary Figure 4: **IV characteristics derived from the thermodynamic model** Output and transfer curves for different voltages resulting from Eq. 10 at room temperature using arbitrary parameters

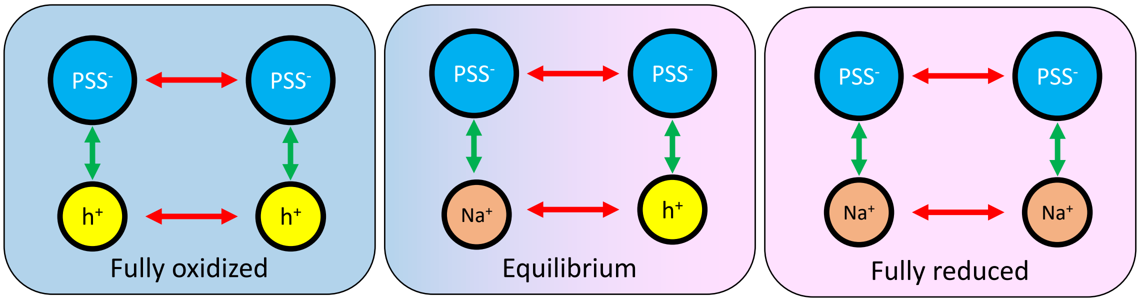

Supplementary Figure 5: **Scheme of the redox process.** The circles indicate the charged species in play, namely PSS^-, holes and sodium cations, and the arrows indicate the Coulomb interaction, either attraction (green) or repulsion (red). Despite the complex chemical process involving multiple species, the energetics of the system does not change dramatically when PEDOT goes from fully oxidized to fully reduced. This explains why an OECT must be described in terms of entropy, and why enthalpy play a secondary role, marking a strong contrast with capacitive systems such as FETs.

Supplementary Note 1: Gibbs free energy of the electrolyte

The Gibbs free energy of the electrolyte:OMIEC system is given by the sum of their free energy G_e and G_o, respectively. Before the OMIEC is immersed in the electrolyte, the electrolyte has a free energy $G_{e,1}$. When the OMIEC is immersed, some ions migrate from the bulk electrolyte into the polymer, effectively diluting the electrolyte and bringing the electrolyte to a free energy $G_{e,2}$. Upon dilution, the difference in energy is given by

$$G_{e,2} - G_{e,1} = \Delta G_e = (H_{e,2} - TS_{e,2}) - (H_{e,1} - TS_{e,1}) = RT \ln \frac{c_1}{c_2} \qquad \text{(Supplementary Eq. 1)}$$

where c_1 and c_2 are the concentrations before and after the OMIEC has been immersed. The enthalpy of the solution depends on the ion concentration (although it is a good approximation to neglect it because of the low ion concentration 100 mM i.e., ideal solution). When the OMIEC is immersed, roughly 10% of its charged moieties are counterbalanced by ions from the electrolyte (Figure 2 in the main text). Let's assume PEDOT:PSS has a PSS density (hence a maximum charge carrier density) of $1\text{x}10^{20}$ cm^{-3} (upper limit of a highly doped semiconductor, PEDOT:PSS is probably much less). By using a film of size 200 μm x 50 μm x 100 nm, one finds that $1\text{x}10^5$ charges move from the electrolyte to the organic film.

For these experiments, the OMIECs were immersed in a beaker (V$\approx$ 1 l) containing the electrolyte. Such volume contains $6.2\text{x}10^{22}$ ions. Often, OECT measurements are performed by casting a drop of electrolyte on top of gate and channel. Considering a volume of 1 ml, the calculation yields $6.2\text{x}10^{19}$ ions.

Therefore, after subtracting the above-calculated number of ions that diffuses in the film, one finds that $c_1 \approx c_2$. Thus $\Delta G_e = 0$. Therefore, the thermodynamics of the electrolyte is not altered by the presence of such a miniaturized thin film. We conclude that the Gibbs free energy of the electrolyte should be counted, but it adds up to the free energy of the polymeric system as a constant. As such, when measuring variation of energy (ΔG), or when we do the derivative to calculate the chemical potential μ, such a constant is lost. It is therefore physically meaningful and mathematically correct to carry out the thermodynamics analysis on the thin film only.

Supplementary Note 2: calculation of the drain current

In order to find the number of charges accumulated in the channel by the gate voltage, Ohm's law (Eq. 7) must be integrated over the channel length from 0 to L (or equivalently from V_{ds} to 0) as done typically for thin-film transistors.

$$\int_0^L I_d = eV_{ds}\Lambda_h \frac{Wt}{L}[PSS^-]\int_0^L (1-\phi(\mu))d\ell \qquad \text{(Supplementary Eq. 2)}$$

yielding

$$I_d = -e\Lambda_h \frac{Wt}{L}[PSS^-]\int_{V_{ds}}^0 (1-\phi(\mu))dV_{ch} \qquad \text{(Supplementary Eq. 3)}$$

after considering a linearly dropping potential V_{ch} along the channel length (direction ℓ)

$$V_{ch}(\ell) = V_{ds}(1-\frac{\ell}{L}) \qquad \text{(Supplementary Eq. 4)}$$

and its differential:

$$dV_{ch} = -\frac{V_{ds}}{L}d\ell \qquad \text{(Supplementary Eq. 5)}$$

In doing so we are assuming i) that the gradual channel approximation is valid *i.e.,* the field across the channel/electrolyte interface is much higher than the field between source and drain across the

channel, and ii) a linearly dropping potential across the channel. The former holds true thanks to the exponential decay of the field in the electrolyte. The second is not necessarily true and strongly depends on the gate voltage. However, the result of the integral is not expected to vary much depending on the charge distribution.
To solve Eq. Supplementary Eq. 3, we need $\phi(\mu)$, that can be found by inverting $\mu(\phi)$ in Eq. 8:

$$\phi(\mu) = (Z\exp(-\frac{\mu}{k_BT}) + 1)^{-1} \qquad \text{(Supplementary Eq. 6)}$$

where $Z = \exp(\frac{\mu^{p^+} - \mu^{p^0}}{k_BT})$. The application of a gate potential has the effect of shifting the system from its chemical equilibrium. By introducing the electrochemical potential $\bar{\mu}$, μ becomes a function of the potentials applied as $e(V_{gs} - V_{ch})$ as explained in the main text.

Combining Supplementary Eq. 3 and Supplementary Eq. 6

$$I_d = -e\frac{Wt}{L}\Lambda_h[PSS^-]\int_{V_{ds}}^{0} dV_{ch}(1 - \frac{1}{Ze^{-\beta(V_{gs}-V_{ch})} + 1}) = e\frac{Wt}{L}\Lambda_h[PSS^-]\frac{1}{\beta}\ln(\frac{Ze^{\beta V_{ds}} + e^{\beta V_{gs}}}{Z + e^{\beta V_{gs}}}) \qquad \text{(Supplementary Eq. 7)}$$

with $\beta = \frac{e}{k_BT}$ (elementary charge divided by thermal energy).

Thus, the current that flows in the channel of an OECT, assuming no enthalpy of mixing, depends on V_{ds}, V_{gs}, and T as follows:

$$I_d = \Lambda_h\frac{Wt}{L}[PSS^-]k_BT\ln(\frac{Ze^{-\beta(V_{gs}-V_{ds})} + 1}{Ze^{-\beta V_g} + 1}). \qquad \text{(Supplementary Eq. 8)}$$

Accordingly, the transconductance $g_m = \frac{dI_d}{dV_{gs}}$ is

$$g_m = e\Lambda_h[PSS^-]\frac{Wt}{L}Z(\frac{1}{e^{\beta V_{gs}} + Z} - \frac{1}{e^{\beta(V_{gs}-V_{ds})} + Z}) \qquad \text{(Supplementary Eq. 9)}$$

and has the typical (and peculiar) bell-shaped form of $g_m(V_{gs})$ of OECTs.
For the transfer measurements in Fig. 4 with $V_{ds} = 5mV$, we exploit the fact that $V_{gs} - V_{ch} \approx V_{gs}$ in order to rewrite Supplementary Eq. 3 as

$$I_d = -e\Lambda_h\frac{Wt}{L}[PSS^-]\int_{V_{ds}}^{0}(1 - \phi(V_{gs} - V_{ch}))dV_{ch} \approx \Lambda_h\frac{Wt}{L}[PSS^-]V_{ds}(1 - \phi(V_{gs})) \qquad \text{(Supplementary Eq. 10)}$$

from which the material-specific property $\phi(\mu)$ can be extracted.

Enhancement/accumulation mode OECTs

For an enhancement mode OECT, the FET model would foresee the following I-V relationship

$$I_d = \frac{Wt}{L}\Lambda_h e\rho_0\frac{V_{gs} - \frac{V_{ds}}{2}}{V_p}V_{ds} \qquad \text{(Supplementary Eq. 11)}$$

Eq. 7 becomes

$$I = \Lambda_h\rho\frac{Wt}{L}V = \Lambda_h(T)\rho_0\phi(T)\frac{Wt}{L}V \qquad \text{(Supplementary Eq. 12)}$$

and Eq. 10 becomes

$$I_d = e\Lambda_h\frac{Wt}{L}\rho_0(V_{ds} - \frac{k_BT}{e}\ln(\frac{Ze^{-\beta(V_{gs}-V_{ds})} + 1}{Ze^{-\beta V_{gs}} + 1})). \qquad \text{(Supplementary Eq. 13)}$$

Gate-channel coupling

A final note on the gate voltage: in order to fully capture the current dependency on the applied gate voltage V_{gs} one must take into consideration that the gate voltage applied differs from the effective gate voltage $V_{g,eff}$ "felt" by the channel that drives the device at the channel/electrolyte interface due to the drop at the gate/electrolyte interface and across the solution. In this work, since we focused on the modeling of the material parameters, we simplified it as

$$V_{gs,\text{eff}} = \alpha V_{gs} \qquad \text{(Supplementary Eq. 14)}$$

where $0 < \alpha < 1$ is the remaining percentage of potential applied after dropping across the electrolyte and at the gate/electrolyte interface. Although the voltage drop from gate/electrolyte interface to electrolyte/PSS interface can have a more complex, nonlinear, voltage-dependent and concentration-dependent, we use a linear approximation for the sake of simplicity, and may be the root of the unmatching fit in Fig. 3 in the turning-off region. A detailed analysis of the voltage drop at the gate/electrolyte and electrolyte/channel interface is given by Romele *et al.* [25].

Supplementary note 3: MATlab code for plotting the channel current

```
x=linspace(0,1,1000);
mu1mu2=1e-21;
g0=1;
alpha=1;
T=300;
kb=1.38e-23;
h1=1e-22;
mu=6.2e+18/alpha*(kb*T.*log(x./(1-x)) + mu1mu2 +2*h1.*x);
vg_new=linspace(-1,1, 1000);
id_new=interp1(mu(2:end-1),x(2:end-1),vg_new);
naan=find(isnan(id_new)==0);
id_new(1:naan(1))=0;
id_new(naan(end):end)=1;
dvg=vg_new(2)-vg_new(1);
vd=-.1;
for sweep=1:length(vg_new)
[yy,idx1]=min(abs(vg_new(sweep)-vg_new));
[yy,idx2]=min(abs(vg_new(sweep)-vd-vg_new));
q(sweep)=sum(id_new(idx1:idx2))/(idx2-idx1+1);
end
plot(vg_new,g0*vd*(1-q));
```

Solvent	α	Error α	h_1	Error h_1	h_2	Error h_2	h_3	Error h_3
ine Water:NaCl	0.087	0.002	0.265	0.037	0.174	0.004	0.219	0.011
Water:KCl	0.103	0.036	0.161	0.026	0.082	0.039	0.122	0.002
EMIM-EtSO$_4$	0.095	0.018	0.229	0.088	0.259	0.103	0.244	0.023
Butanol:LiClO$_4$	0.199	0.044	0.253	0.082	0.258	0.091	0.255	0.096
Water:LiClO$_4$	0.136	0.046	0.028	0.009	0.044	0.004	0.036	0.002
ine								

Supplementary Table 1: Values and errors resulting from the fit of the transfer curves in Fig. 4. The experimental I-V transfer curves are transformed into a $\mu - \phi$ as explained in as explained in Supplementary Note 2. This is possible only because the curves are measured at vanishing drain voltage. By integrating the chemical potential, the Gibbs free energy is obtained, which is the curve on which the fit is carried out.
